# Tumor microenvironment immune subtypes for classification of novel clear cell renal cell carcinoma profiles with prognostic and therapeutic implications

**DOI:** 10.1097/MD.0000000000024949

**Published:** 2021-03-19

**Authors:** Qiang Wang, Jinding Hu, Weiting Kang, Jin Wang, Yuzhu Xiang, Min Fu, Hui Gao, Zhilong Huang

**Affiliations:** a Department of Human Resources, Shandong Provincial Hospital Affiliated to Shandong First Medical University; b Department of Human Resources, Shandong Provincial Hospital, Cheeloo College of Medicine, Shandong University, Jinan; c Department of Urology, The Second People's Hospital of Liaocheng; d Department of Urology, The Second Hospital of Liaocheng Affiliated to Shandong First Medical University, Liaocheng; e Department of Urology, The First Affiliated Hospital of Shandong First Medical University; f Department of Urology, Shandong Provincial Qianfoshan Hospital, Cheeloo College of Medicine, Shandong University; g Department of Urology, Shandong Provincial Hospital, Cheeloo College of Medicine, Shandong University; h Department of Urology, Shandong Provincial Hospital Affiliated to Shandong First Medical University, Jinan; i Department of Urology, Liaocheng People's Hospital, Liaocheng, Shandong 250000, China.

**Keywords:** clear cell renal cell carcinoma, clinical prognosis, genetic diversity, immune subtypes, tumor microenvironment

## Abstract

Currently, no effective prognostic model of clear cell renal cell carcinoma (ccRCC) based on immune cell infiltration has been developed. Recent studies have identified 6 immune groups (IS) in 33 solid tumors. We aimed to characterize the expression pattern of IS in ccRCC and evaluate the potential in predicting patient prognosis. The clinical information, immune subgroup, somatic mutation, copy number variation, and methylation score of patients with TCGA ccRCC cohort were downloaded from UCSC Xena for further analysis. The most dominant IS in ccRCC was the inflammatory subgroup (immune C3) (86.5%), regardless of different pathological stages, pathological grades, and genders. In the C3 subgroup, stage IV (69.1%) and grade 4 (69.9%) were the least presented. Survival analysis showed that the IS could effectively predict the overall survival (OS) (*P* < .0001) and disease-specific survival (DSS) (*P* < .0001) of ccRCC alone, of which group C3 (OS, HR = 2.3, *P* < .001; DSS, HR = 2.84, *P* < .001) exhibited the best prognosis. Among the most frequently mutated ccRCC genes, only VHL and PBRM1 were found to be common in the C3 group. The homologous recombination deficiency score was also lower. High heterogeneity was observed in immune cells and immunoregulatory genes of IS. Notably, CD4+ memory resting T cells were highly infiltrating, regulatory T cells (Treg) showed low infiltration, and most immunoregulatory genes (such as CX3CL1, IFNA2, TLR4, SELP, HMGB1, and TNFRSF14) were highly expressed in the C3 subgroup than in other subgroups. Enrichment analysis showed that adipogenesis, apical junction, hypoxia, IL2 STAT5 signaling, TGF-beta signaling, and UV response DN were activated, whereas E2F targets, G2M checkpoint, and MYC targets V2 were downregulated in the C3 group. Immune classification can more accurately classify ccRCC patients and predict OS and DSS. Thus, IS-based classification may be a valuable tool that enables individualized treatment of patients with ccRCC.

## 1 Introduction

Kidney cancer is one of the most common cancers affecting the urogenital system. It is estimated that in 2020, approximately 73,750 new cases of kidney cancer cases and more than 14,830 kidney cancer deaths will occur in the United States.^[1]^ Renal cell carcinoma (RCC) is the most common type of kidney cancer and accounts for 85% of kidney cancer cases. It is a group of heterogeneous malignancies originating from renal tubular cells.^[2]^ RCC is further divided into clear cell renal cell carcinoma (ccRCC), which makes up 75% to 80% of RCCs, papillary cell carcinoma that comprises 15% of RCCs, and chromophobe cell carcinoma that makes up 5% of RCCs.^[3]^ Staging RCC is based on size, position, and lymph node involvement.^[4,5]^ The higher the stage, the worse the prognosis. A stage I or II tumors is enclosed wholly in the kidney. Stage III tumors can extend into major veins or adrenal glands within Gerota's fascia or can involve 1 regional lymph node involvement. Stage IV tumors can invade beyond Gerota's fascia and/or have distant metastases.^[5]^ Grade is another most powerful prognostic factors in patients with RCC. The Fuhrman grading system is currently most widely used by pathologists in Europe and the United States. The 5-year survival of stage IV RCC was as low as 20%.^[5]^ Five-year overall survival rates were 64%, 34%, 31%, and 10% for grades 1 through 4, respectively.^[6]^ Therefore, there is an urgent need to better understand the mechanisms driving RCC development and progression for better diagnostic and therapeutic tools.

To date, most RCC research has focused on the ccRCC subtype. Greater than 90% of ccRCC cases are characterized by loss of heterozygosity on the short arm of chromosome 3.^[7]^ About 50% of the cases have a genetic mutations^[7,8]^ while 5% to 10% of the cases are characterized by promoter hypermethylation.^[8]^ These alterations lead to high-frequency inactivation of the biallelic VHL. Loss of VHL function stabilizes hypoxia-inducible factor 1α (HIF1α) and HIF2α, thereby enhancing expression of hypoxia-responsive genes, such as the vascular endothelial growth factor (VEGF) family and platelet-derived growth factor (PDGF) family that promote angiogenesis.^[7]^ Based on the role of the VHL-HIF axis in ccRCC, various drugs have been developed to target this signaling pathway, including the tyrosine kinase inhibitors sunitinib, sorafenib, pazopanib, and axitinib that target the VEGF and PDGF receptors, bevacizumab, which binds and inhibits VEGF, as well as everolimus and temsirolimus that inhibit rapamycin (mTOR) signaling. In addition, genes involved in chromatin modification, including PBRM1, SETD2, KDM5C 9, KDM6A9, and BAP1,^[8–10]^ are also frequently mutated in ccRCC. Despite the above genetic factors being druggable targets, the median survival time of metastatic RCC remains low, at 11 to 26 months,^[11–14]^ highlighting an urgent need for novel treatment options for RCC prognosis.

Cancer development and progression is the product of complex interactions between tumor cells and their microenvironment. Tumor infiltrating immune cells can modulate ccRCC progression and may have prognostic value. CD4+ T cells modulate the proliferation of RCC via the TGFβ1/YBX1/HIF2α signaling axis.^[15]^ In many cancers, including RCC, high levels of activated CD8+ T cells are associated with better prognosis.^[16,17]^ Regulatory T cells (Tregs), which secrete immunosuppressive cytokines, cause T cell dysfunction,^[18,19]^ while tumor-associated macrophages may promote or suppress tumor development.^[20,21]^ A variety of immune cells form a network of regulatory systems in tumors through complex interactions, thereby regulating cancer development and progression. To further understand the role of immune cells in tumors, Thorsson et al developed a novel solid tumor global immune classification system based on transcriptomic analyses of 33 solid tumors and identified 6 different immune subtypes (ISs).^[22]^ The wound healing (C1) subtype is characterized by elevated levels of angiogenic genes, high proliferative rates, and low Th1/Th2 cell ratios. The IFNγ dominant (C2) subtype exhibits high proliferation rates, the highest intratumoral heterogeneity, macrophage M1/M2 polarization, diversity of CD8T cell populations, and the largest T cell receptor (TCR) diversity. The inflammatory (C3) subtype exhibits elevated Th17 and Th1 genes, low to moderate proliferation rates, lower aneuploidy levels, higher somatic copy number changes, and the best prognosis. The lymphocyte depleted (C4) subtype is characterized by moderate cell proliferation, intratumor heterogeneity, a prominent macrophage signature with Th1 suppressed and a high M2 response, and is associated with poor prognosis. The immunologically quiet (C5) subtype exhibits the lowest lymphocyte response and highest macrophage response, mainly M2, with lower value-added and heterogeneity. The TGF-b dominant (C6) subtype consists of a group of mixed tumors with the highest TGF-β signal and a high lymphocytic infiltrate and a balanced Th1:Th2 ratio. Subtype C6 and C4 are associated with the worst prognosis.

This immune classification spans traditional cancer classifications based on anatomical site of origin and suggests that there are treatments that can be considered, regardless of the location or histology of the tumor. However, the proportion of these subtypes and their impact on prognosis varies widely between tumor subtypes. Here, we evaluated the immune subtypes of ccRCC and the potential role of immune group (IS) in ccRCC. We analyzed clinical, genomics, and transcriptomic data to develop a cancer immune map and a theoretical basis for the use of immunotherapy against ccRCC.

## 2 Materials and methods

### 2.1 Data download

We used the R package “UCSCXenaTools” to download gene expression RNAseq (TOIL RSEM tpm), phenotype (Immune subtype, Curated clinical data), somatic mutation (gene level non-silent mutation), DNA methylation (Methylation450K), signatures (HRD score, genome-wide DNA damage footprint), and copy number (gistic2_thresholded) data of pan-cancer dataset (including 9204 cases) from the UCSC Xena data center on March 8, 2020. Next, we analyzed a ccRCC dataset (including 515 cases) was obtained from the pan-cancer research cohort.^[23]^ The UCSC Xena data center is a collection of public databases, including TCGA, ICGC, TARGET, GTEx, and CCLE.^[24]^ The database is standardized so it can be combined, linked, filtered, browsed, and downloaded.

### 2.2 Data preprocessing

Before analyses of the downloaded RNAseq, HRD score, and DNA methylation data from ccRCC patients, limma, an R package was used to correct for batch effect. Heatmaps were generated using the R package “pheatmap” and graphs plotted using the R package “ggplot2.” ccRCC somatic mutations were defined as either being non-silent or wild type. Statistical analyses were used to establish the proportion of patients with non-silent mutations in each immune subtype group. Copy number variations (CNV) can consist of a deletion (homogeneous deletion and single-copy deletion), normal copy, amplification (low-level copy number and high-level copy number amplification). The following information was extracted from ccRCC phenotype data: immunotype information, pathological stage, pathological grade, gender, and prognosis. The proportions of the pathological stage, pathological grade, and gender in IS were statistically analyzed and presented as balloon plots using “ggplot2.” Survival analysis was done using the R packages “survival” and “survfit.” Next, cases with complete clinical information, including age, gender, pathological stage, and pathological grade were selected for multivariate COX regression analysis. Immune subtype was classified into subgroups C3 and non-C3. Age was divided into 2 categories: <60 years old and ≥60 years old. Cancer stage was either stage I, stage II, stage III, or stage IV. Pathological grade was either G1, G2, G3, and G4.

### 2.3 Immune infiltration analysis

The CIBERSORTx,^[25]^ online tool was used to analyze the degree of infiltration by immune cells into different samples. Gene expression data was analyzed to establish gene expression profiles and to estimate abundance of various cell types within mixed cell populations. The 22 immune cell types (LM22) were used as a signature matrix file. RNA expression profile was used as the mixture file, and the B-mode selected for batch correction. The disable quantile normalization was checked, as well as the run in absolute mode. Permutation for significance analysis is defined as 1000. The R packages “pheadmap” and “ggplot2” was used to draw heatmaps and boxplots, respectively.

### 2.4 ssGSEA and GSEA analysis

Single-sample Gene Set Enrichment Analysis (ssGSEA) analysis was done on the batch-corrected RNA expression profiles. The ssGSEA score was calculated using the Bioconductor's gsva package on R. Molecular signatures were downloaded from MSigDB, and the gene sets selected as H: hallmark gene sets. Batch-corrected RNA expression profiles were used for Gene Set Enrichment Analysis (GSEA) analysis.^[26]^ Heatmaps were drawn using the R package “pheadmap.”

### 2.5 Statistical analysis

Statistical analyses were done using the R software (version 3.6.1; https://www.R-project.org), statistical significance was set at *P* < .05. Survival data was analyzed using the Kaplan–Meier curve while analysis of the factors that affected patient survival was done using univariate COX regression. The multivariate COX regression analysis was used to determine independent prognostic factors.

## 3 Results

### 3.1 Distribution characteristics of IS in ccRCC patients’ clinical data

**Figure 1:**
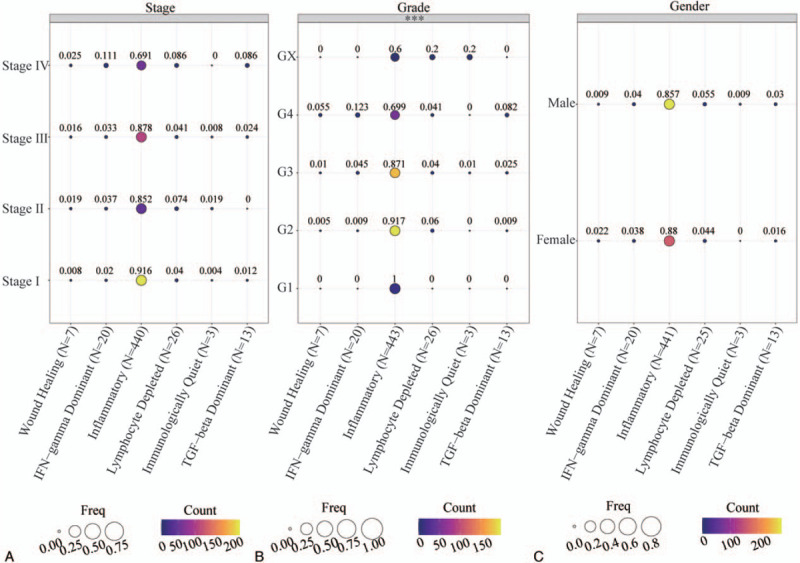
Distribution of IS across different clinical features of patients with ccRCC. (A) A bubble chart showing the distribution of IS in the pathological stage. (B) A bubble chart showing the distribution of IS in the pathological grade. (C) A bubble chart showing gender distribution characteristics of IS. The size of the circle represents the proportion of IS in different clinical characteristics, and the colors represent the number of patients. ^∗∗∗^*P* < .001. ccRCC = clear cell renal cell carcinoma, IS = immune group.

We collected a total of 512 ccRCC patients with immunophenotyping, 183 females, and 329 males. Among them, there were 509 patients with pathological staging data and 512 patients with pathological grading data. We analyzed the distribution of IS in different subgroups. This analysis revealed that in pathological stage of ccRCC, the 6 ISs exhibited an identical distribution pattern (Fig. 1A). Among patients at different pathological stages, the inflammatory subtype (Immune C3) predominates, ranging from 69.1% in stage IV to 91.6% in stage I. In clinical stage IV, the proportion of IFN-γ dominant (Immune C2) was >10%, and the proportion of immune subtypes (C1, C2, C4, C5, C6) in the other stages was <10% (Fig. 1A). The ISs occur at different ratios at different pathological grades (*P* < .001; Fig. 1B). Similarly, at different pathological grades, the immune C3 subtype gradually recreasing from 69.9% in grade 4 to 100% in grade 1 (Fig. 1B). Among ccRCC cases at the grade of 4, IFN-γ dominant (immune C2) accounts for >10%, with the proportion of the remaining ISs being <10% (GX classification excluded). IS analysis in gender, revealed that it has the same composition ratio in males and females (Fig. 1C) and the inflammatory (immune C3) subtype predominates. Immune C3 accounts for 85.7% and 88.0% of the IS in males and females, respectively, with the other subtypes accounting for <10%.

### 3.2 Molecular characteristics of ccRCC in different IS

**Figure 2:**
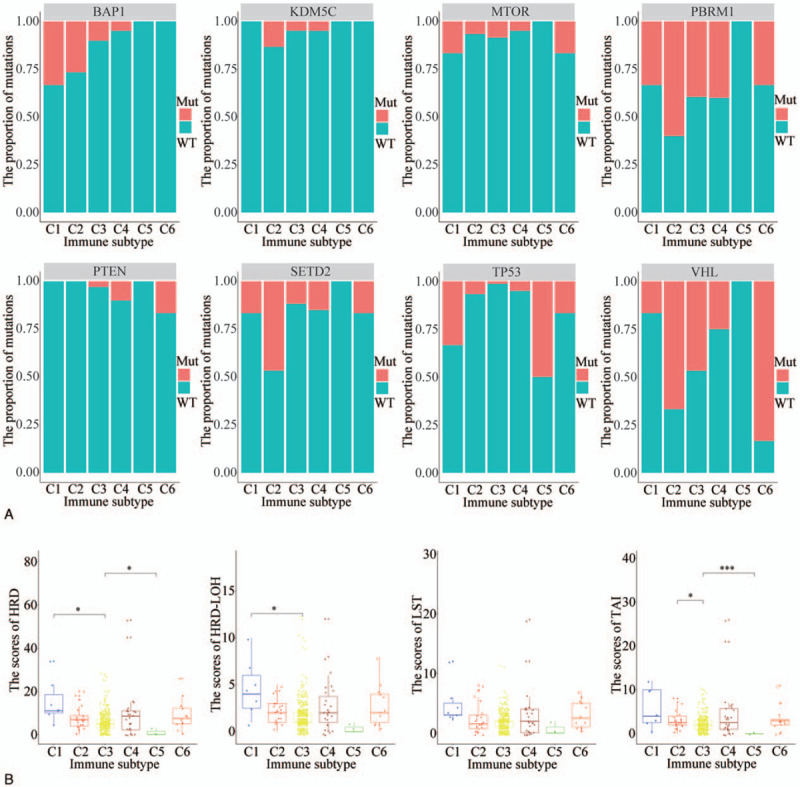
Distribution characteristics of high-frequency mutant genes and homologous recombination repair defects in ccRCC. (A) The bar graph showing the distribution of high-frequency mutant genes in IS. (B) The boxplot showing the distribution of homologous recombination repair defects in IS. ^∗^*P* < .05, ^∗∗^*P* < .01, ^∗∗∗^*P* < .001. ccRCC = clear cell renal cell carcinoma, IS = immune group.

Next, we analyzed the various immune subtypes for the status of genes that are commonly mutated in ccRCC. This analysis revealed that BAP1 mutations mainly occur in C1 (33.3%), followed by C2, C3, and C4. KDM5C mutation rate is generally low and is highest in C2 (13.3%). In general, the mutation frequency of mTOR and PTEN genes is low. In the C1 and C6 subtype, the mutation frequency of mTOR is the highest, and the mutation frequency of mTOR in the 2 groups is 16.7%. In the C6 group, PTEN had the highest mutation frequency, which was 16.7%. PBRM1 mutation frequency is very high in each IS, except for C5 (0.0%); it is highest in C2 (60%). SETD2 mutations mainly affect C2 at a mutation frequency of 46.7%. TP53 mutations mainly affect C5 (50%) and C1 (33.3%). VHL mutation frequency is very high in all IS, except in C5 (0.0%); its mutation frequency is 83.3%, 66.7%, 46.7%, 25.0%, and 16.7% in C6, C2, C3, C4, and C1, respectively. The mutation frequency of different genes in different IS offers a basis for targeted immunotherapy in ccRCC. The genes with the higher mutation frequencies are BAP1, PBRM1, and TP53 in C1; PBRM1, SETD2, and VHL in C2; PBRM1 and VHL in C3; PBRM1 in C4; and PBRM1 and VHL in C6. TP53 is the only mutated gene in C5 (Fig. 2A).

Next, we evaluated the total scores of homologous recombination repair defects (HRD), loss of heterozygosity (LOH), telomere allele imbalance (TAI), and large-scale state transition (LST) in ccRCC. We observed that relative to C3, HRD score was highest in C1 (*P* < .05) and lowest in C5 (*P* < .05). Differences were not statistically significant in the other groups. Compared with C3, the HRD–LOH score was highest in C1 (*P* < .05) while differences in the other groups lacked statistical significance. Compared with C3, the difference between LST scores in each group was not statistically significant. Compared with C3, the TAI score was highest in C2 (*P* < .05) and lowest in C5 (*P* < .001) and differences in the other groups were not statistically significant (Fig. 2B).

### 3.3 The prognostic value of IS in ccRCC

**Figure 3:**
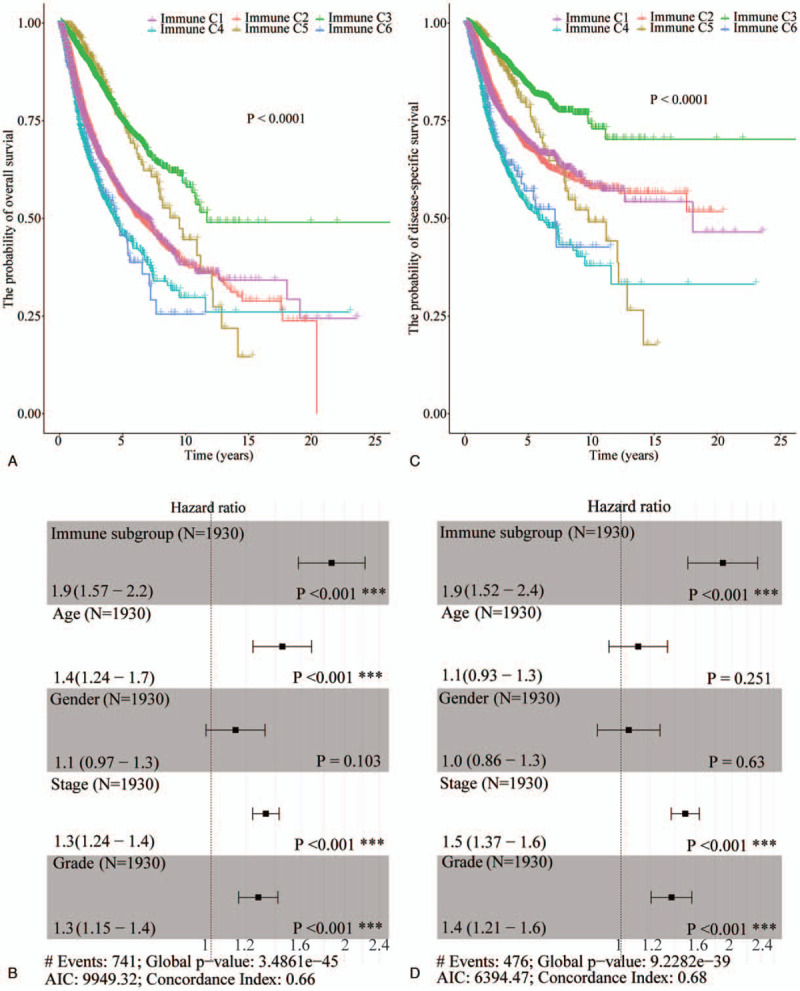
Prediction value of IS for OS and DSS in pan-cancer. (A) The impact of IS on OS of patients with pancancer. (B) Multivariate COX analysis showing the impact of IS (non-C3 vs C3), gender (female vs male), age (>60 yr vs <60 yr), pathological stage, and pathological grade on OS of pan-cancer. (C) The impact of IS on DSS in patients with pan-cancer. (D) Multivariate COX analysis results showing the impact of IS (non-C3 vs C3), gender (female vs male), age (>60 yr vs <60 yr), pathological stage, and pathological grade on DSS of pan-cancer. DSS = disease-specific survival, IS = immune group, OS = overall survival.

Next, we evaluated the impact of different IS on the prognosis of pan-cancer. A total of 9204 patients were included in the study, but the clinical information of some patients was incomplete, such as age, gender, grade, stage, and survival status. After we excluded these patients, 1930 patients remained. The relationship between overall survival (OS) and disease-specific survival (DSS) of patients with pan-cancer and different ISs evaluated. This analysis revealed that in pan-cancer, IS is an effective predictor of cancer prognosis (*P* < .0001) (Fig. 3A). Subtype C3 was associated with the best 10-year OS rate, followed by C5. C1 and C2 had exhibited a similar correlation with prognosis while C4 and C6 correlated with the poorest prognosis. Multivariate COX analysis revealed that IS is an effective predictor of OS in pan-cancer (HR = 1.9, *P* < .001), and is superior to pathological stage (HR = 1.3, *P* < .001) and grade (HR = 1.3, *P* < .001) (Fig. 3B). The 10-year DSS for pan-cancer was found to be similar to its 10-year OS (Fig. 3C and D). However, the DSS in C5 decreases rapidly and the 10-year DSS is lower than C1 and C2. Multivariate COX analysis revealed that IS alone can effectively predict DSS in pan-cancer (HR = 1.9, *P* < .001), and is superior to pathological stage (HR = 1.5, *P* < .001) and grade (HR = 1.4, *P* < .001) (Fig. 3D).

**Figure 4:**
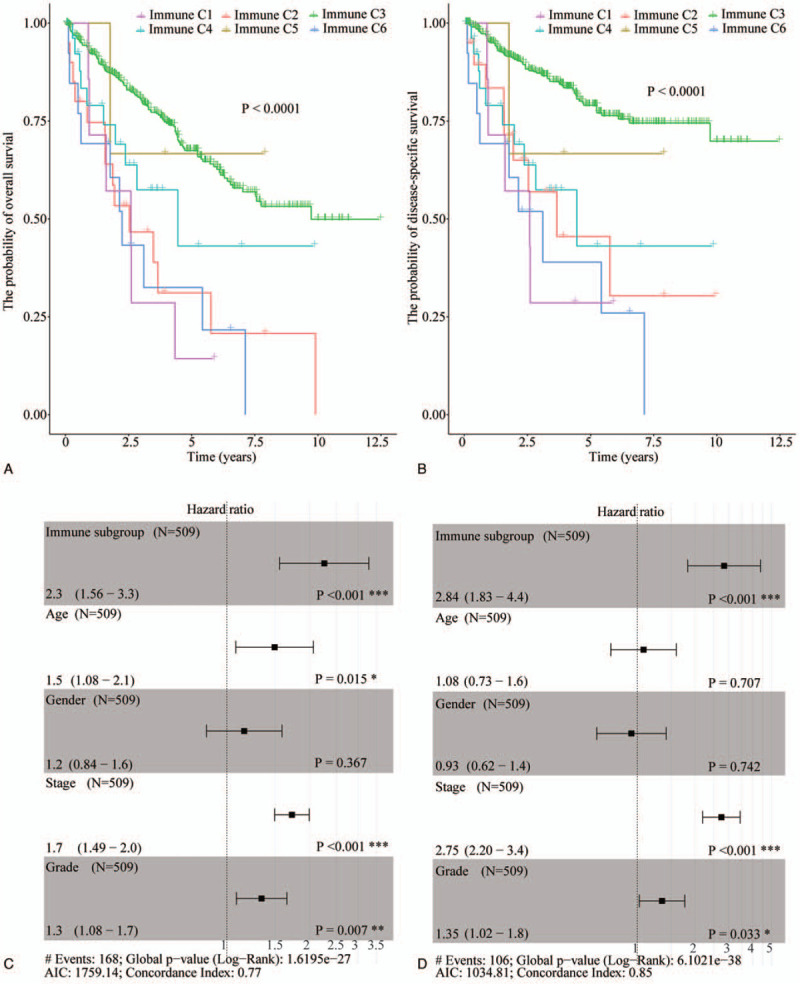
The value of IS on the OS and DSS of ccRCC. (A) Impact of different IS on the OS. (B) Multivariate COX analysis of IS (non-C3 vs C3), gender (female vs male), age (>60 yr vs <60 yr), pathological stage, and pathological grade on the OS of ccRCC. (C) Survival analysis of the value of different IS on the DSS. (D) Multivariate COX analysis of IS (non-C3 vs C3), gender (female vs male), age (>60 yr vs <60 yr), pathological stage, and pathological grade on the DSS of ccRCC. ccRCC = clear cell renal cell carcinoma, DSS = disease-specific survival, IS = immune group, OS = overall survival.

Next, we evaluated the impact of different IS on ccRCC prognosis. To this end, data from 515 ccRCC patients was analyzed for the relationship between ccRCC OS and DSS in different IS. This analysis revealed IS effectively predicts prognosis in ccRCC (*P* < .0001) and C3 correlates with the best OS and DSS relative to the other groups (Fig. 4A and B). Multivariate COX analysis revealed that IS effectively predicts OS (HR = 2.3, *P* < .001) and DSS (HR = 2.84, *P* < .001), and is superior to pathological stage (OS: HR = 1.7, *P* < .001, DSS: HR = 2.75, *P* value < .001) and pathological grade (OS: HR = 1.3, *P* = .007, DSS: HR = 1.35, *P* = .033) (Fig. 4C and D). Like in pan-cancer, IS can be used as an independent predictor of prognosis in ccRCC.

### 3.4 Characteristics of ccRCC infiltration by different IS

**Figure 5:**
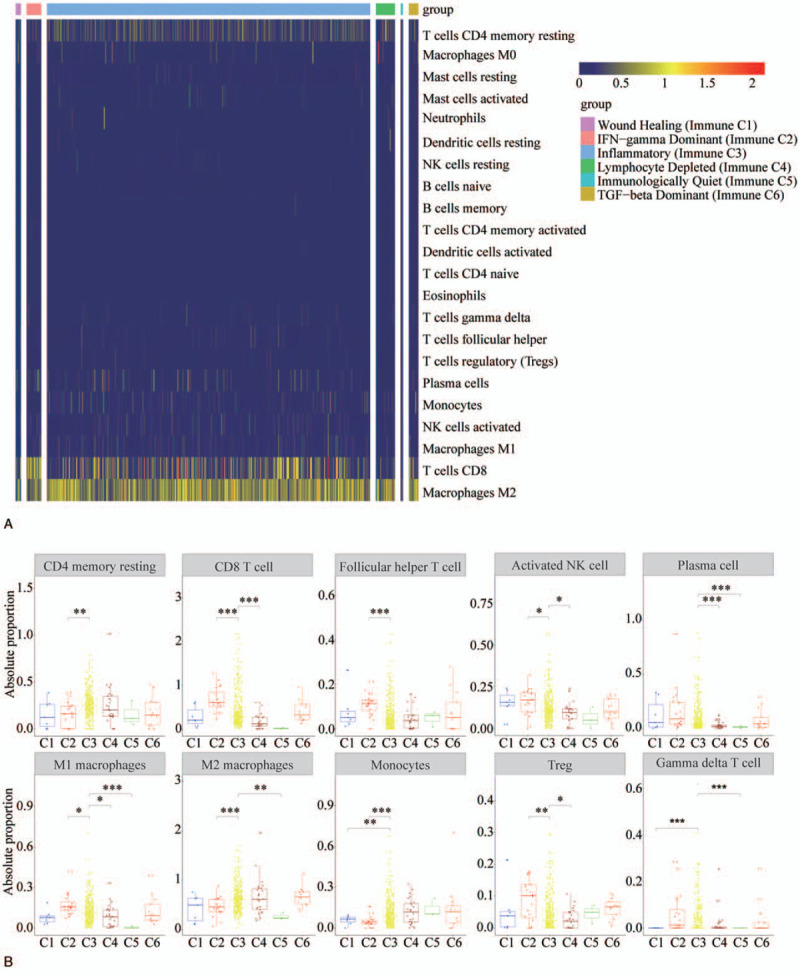
Immune cell infiltration characteristics of different ISs in patients with ccRCC. (A) A heatmap showing the distribution of immune cells of different IS in ccRCC populations. (B) A boxplot showing the distribution of the top 10 abundant immune cells among different IS. ^∗^*P* < .05, ^∗∗^*P* < .01, ^∗∗∗^*P* < .001. ccRCC = clear cell renal cell carcinoma, IS = immune group.

Analysis of different ISs in patients with ccRCC revealed that the immune cell abundance pattern of C3 was significantly different from other groups. M2 macrophages, CD8+ T cells and CD4+ memory resting T cells are the most abundant immune cell types in ccRCC (Fig. 5A). Next, the top 10 most abundant cell types were selected for further analysis. This analysis revealed that relative to subtype C3, monocytes and gamma delta T cells reduced in C1. Compared with subtype C3, CD4+ memory resting T cells, M2 macrophages and monocytes were reduced, CD8+ T cells, follicular helper T cells, activated NK cells, M1 macrophages, and Tregs were elevated in C2. Compared with subtype C3, CD8+ T cells, activated NK cells, plasma cells, M1 macrophages, and Tregs were decreased in C4. Compared with subtype C3, plasma cells, M1 macrophages, M2 macrophages, and gamma delta T cells were reduced in C5. The distribution of immune cells in C6 was the same as the C3 (Fig. 5A and B). The difference between the abundance of immune cells in C3 subtype and other groups (such as C1, C2, C4, C5 subtype) may affect the prognosis of ccRCC.

### 3.5 Expression of immunoregulatory genes in ccRCC ISs

**Figure 6:**
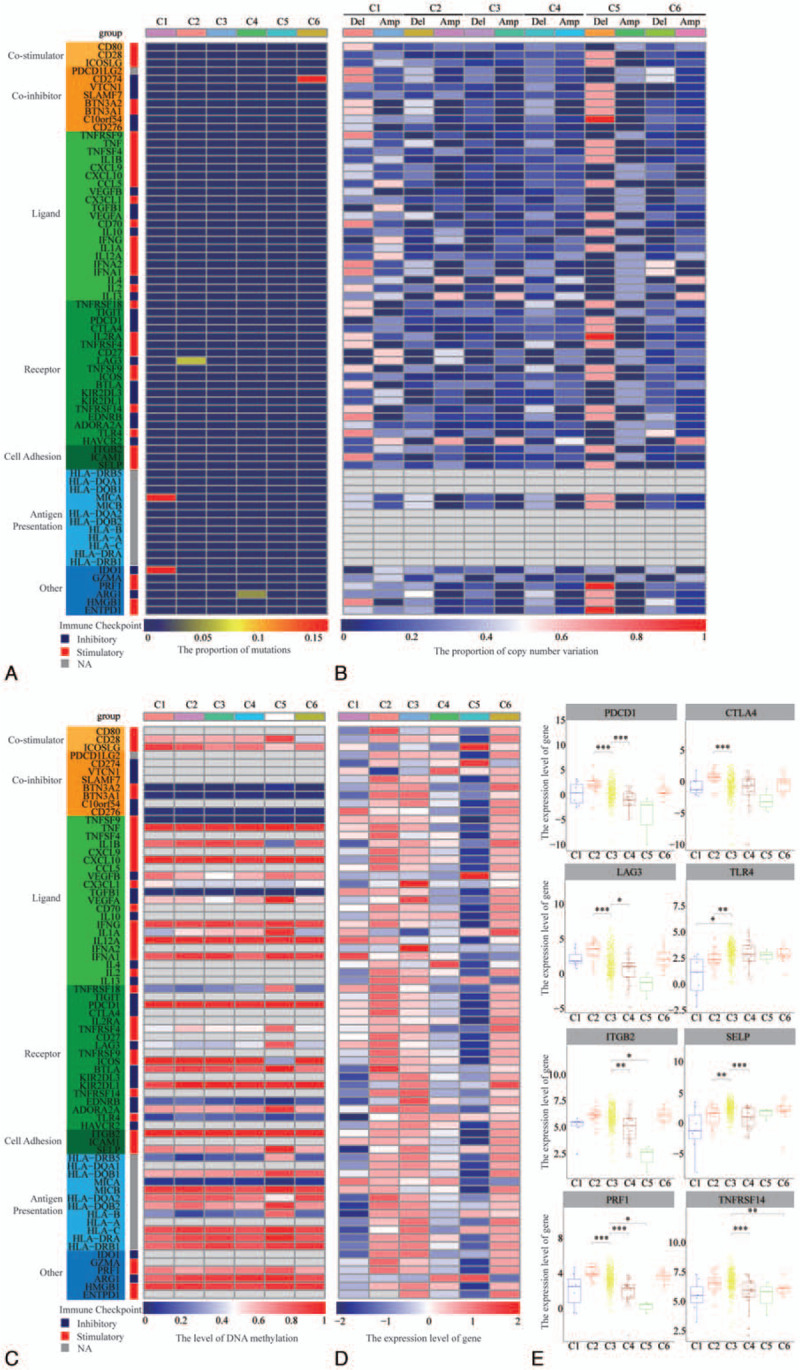
The characteristics of immunoregulatory genes in different IS of patients with ccRCC. (A) Somatic mutations of immunoregulatory genes in different IS. (B) The copy number variations of immunomodulatory genes in different IS. (C) The methylation levels of immune regulatory genes in different IS. (D) Gene expression levels of immunoregulatory genes in different IS. (E) Gene expression levels of common immunomodulatory genes in different IS. ^∗^*P* < .05, ^∗∗^*P* < .01, ^∗∗∗^*P* < .001. ccRCC = clear cell renal cell carcinoma, IS = immune group.

Next, we examined the levels of somatic mutations, copy number variations, methylation, and expression levels of immunoregulatory genes in different ISs. This analysis revealed variations in somatic mutations on immunoregulatory genes in different ISs (Fig. 6A). MICA (16.7%) and IDO1 (16.7%) were significantly mutated in C1, LAG3 was significantly mutated (6.7%) in C2, ARG1 (5.0%) had significant mutations in C4, and CD274 (16.7%) was significantly mutated in C6. The other immunoregulatory genes were not detected in different ccRCC IS.

Analysis of CNV features in immunoregulatory genes varying levels of copy number deletion and amplification in different ISs (Fig. 6B). The frequency of copy number deletions in C1 and C5 groups was significantly higher than in other groups. Deletion rates for C10orf54, IL2RA, PRF1, and ENTPD1 were as high as 100% in C5. The frequency of immune gene copy number amplification in the C1 was significantly higher than in other groups. In C1, C2, C3, C4, and C6 groups, IL4 (42.9–61.5%), IL13 (42.9–61.5%), and HAVCR2 (48–69.2%) exhibited higher copy number deletion rates.

Analysis of methylation revealed that most immunoregulatory genes are highly methylated in different IS of ccRCC, including TNF, CXCL10, IFNG, IL12A, IFNA1, PDCD1, BTLA, KIR2DL1, ITGB2, MICB, HLA-C, HLA-DRA, HLA-DRB1, ARG1, and HMGB1. The degree of methylation of some immunoregulatory genes in the C5 was significantly higher than in other subgroups, while the degree of methylation on VEGFB and VEGFA in C3 was significantly lower than in other subgroups (Fig. 6C).

The gene expression analysis indicated that most immunoregulatory genes are highly expressed in C2, C3, and C6, and that expression CX3CL1 and IFNA2D expression is significantly elevated in C3. In C5, most immunoregulatory genes were downregulated, while ICOSLG, CD274, VEGFB, and ARG1 were significantly increased (Fig. 6D). Relative to C3, TLR4 was reduced in C1. While SELP was decreased, PDCD1, CTLA4, LAG3, TLR4, and PRF1 were increased in C2. PDCD1, LAG3, ITGB2, SELP, PRF1, and TNFRSF14 were decreased in the C4. ITGB2 and PRF1 were decreased in the C5, while TNFRSF14 was decreased in the C6 (Fig. 6E).

### 3.6 Analysis of enrichment pathways of different immune subgroups in ccRCC

**Figure 7:**
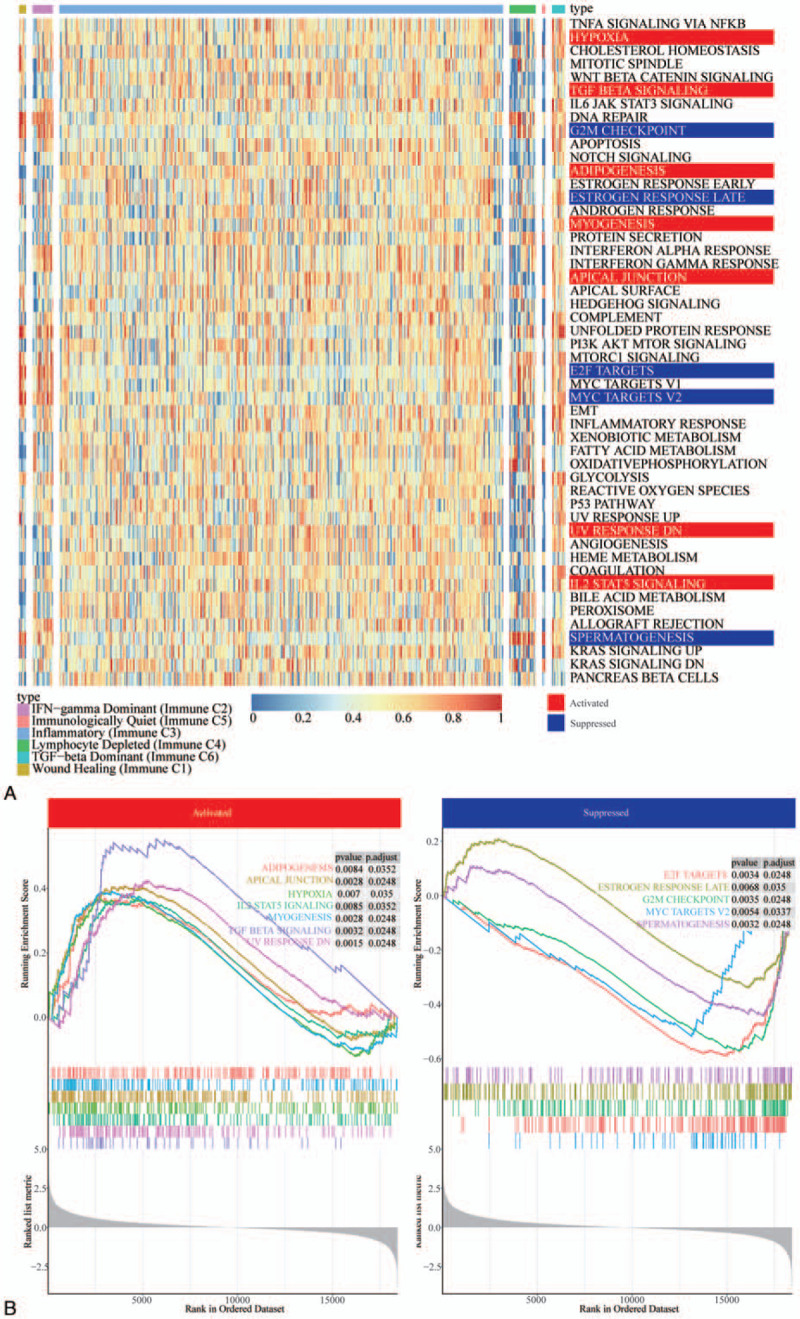
Pathway enrichment analysis for upregulated and downregulated immune subtypes. (A) ssGSEA analyzes the cellular signaling pathways in different IS. (B) GSEA analyzes the activated and inhibited cellular signaling pathways in the C3 and other subgroups. IS = immune group.

Next, we carried out ssGSEA pathway analysis in different ISs. This analysis revealed cellular pathway variations between C3 (which exhibited the best prognosis) and the immune subgroups with poor prognosis (C1, C2, C4, C5, and C6) (Fig. 7A and B). Relative to the IS with poor prognosis, activated pathways in C3 included adipogenesis, apical junction, hypoxia, IL2 STAT5 signaling, myogenesis, TGF-beta signaling, and UV response DN. Suppressed pathways in C3 included E2F targets, estrogen response late, G2M checkpoint, MYC targets V2, and spermatogenesis.

## 4 Discussion

Renal cell carcinoma (RCC) is the most common form of kidney cancer. It is categorized into various subtypes, with ccRCC representing about 85% of all RCC.^[27]^ ccRCC is characterized by low sensitivity to chemoradiotherapy, which creates an urgent need for novel and effective treatment options. The study found RCC is immune-sensitive cancer that widely expresses PD-L1, a factor that is associated with poor prognosis.^[28–30]^ Relative to inhibitor of mammalian target of rapamycin (mTOR, a major breakthrough in ccRCC treatment), the PD-1 blocker nivolumab was found to improve OS (25 months vs 19.6 months).^[31,32]^ Novel immune checkpoint inhibitors are also being evaluated in RCC. For example, IMP-321, an APC activator targeting LAG3, has been clinically tested in metastatic RCC.^[33]^ Although immune checkpoint inhibitors have demonstrated significant benefits against RCC, they have some drawbacks. Combined use of nivolumab and the CTLA-4 blocker, ipilimumab, in the treatment of metastatic RCC elicits response in only 43% of patients, while triggering adverse effects in 88% of patients.^[34]^ PD-L1 expression may be a useful biomarker for PD-1/PD-L1 inhibitory response. However, it has been found that as many as 18% of patients with PD-L1 negative tumors respond to treatment,^[35]^ while many patients with PD-L1 positive tumors do not respond.^[29,36]^ Given the high number of immunotherapies under development, it is crucial that the most appropriate therapy for each patient is selected.

Currently, no effective ccRCC prognostic tools based on immune cell infiltration are in clinical use. Thorsson et al recently developed a whole-cancer immune classification system that covers 33 solid cancer. This system consists of 6 ISs with unique immune and genomic features as well as clinical outcomes.^[22]^ However, these ISs vary significantly between cancer types. Here, we analyzed TCGA datasets to identify ISs in ccRCC patients. We evaluated the differences in clinical treatment, grouping patterns, and immune infiltration in the context of various ISs in ccRCC. Immune infiltration status offers basis for targeted immunotherapy in ccRCC. Our analysis revealed that IS C3 predominates in patients at varying ccRCC pathological stages, grades, and gender. Compared with other pathological stages, the proportion of C3 in stage IV was significantly lower. Relative to other pathological grades, advancing pathological grade correlated with a reduction in the C3 proportion. Survival analysis and multivariate COX analysis showed that IS has a high capacity to predict survival and is an independent predictor of ccRCC prognosis. IS can use immune infiltration and molecular patterns instead of tumor location or histology to stratify patients to predict the prognosis of patients, which can help clinicians personalize patient care.

Our research has found that patients with C3 subtype have the best prognosis. Through various analyses, including gene mutations, immune-related gene expression and immune cell composition, we can infer some of the underlying mechanisms for C3 subtype to have the best prognosis. The mutation frequency of mutation-prone genes in ccRCC was differentially distributed in different ISs, providing a theoretical basis for differential prediction of prognosis by different subgroups and the use of combined therapy targeting various ISs. The mutation frequency of the VHL gene was lower in the C3 subtype than that of C2 and C6 subtypes. The mutation frequency of the TP53 gene was lower in the C3 subtype than that of all other subtypes. In the C3 subtype, the mutation frequency of the BAP gene was lower than that of C1 and C2 subtypes. In the C3 subtype, the mutation frequency of PBRM1 gene was lower than that of C2 subtype. Study found most sporadic ccRCC cases are characterized by the loss of function of the von Hippel-Lindau (VHL) tumor-suppressor gene.^[37]^ The inactivation of VHL results in accumulation of hypoxia-inducible factors (HIFs) and overexpression of many genes, including those that promote angiogenesis and reprogramming of cellular metabolism.^[37]^ The importance of the TP53 gene as a tumor suppressor is highlighted in human cancer where it is the most commonly mutated gene, with mutations found in a broad variety of cancer types.^[38–40]^ The loss of the remaining PBRM1 or BAP1 allele can be associated with different ccRCC grades and aggressiveness.^[10,41]^ Therefore, the low frequency mutation of VHL, TP53, PBRM1, and BAP1 may be a potential mechanism for the better prognosis of the C3 subtype. The difference of gene mutation frequency in different immune subgroups suggests whether we can consider corresponding targeted drugs combined with immunosuppressive agents. Of course, these speculations require further prospective clinical trials. In addition, the difference in the level of immune cells infiltration can also explain the better prognosis of the C3 subtype. Compared with C1 subtype, the monocytes increased in the C3 subtype. Compared with C2 subtype, the CD4 memory resting cells and monocytes increased, while the regulatory T cell (Treg) cells decreased in the C3 subtype. Compared with the C4 subtype, CD8T cells, activated NK cells, plasma cells, and M1 macrophages increased in the C3 subtype. Compared with C5 subtype, plasma cells, M1 macrophages, and gamma delta T cells increased in the C3 subtype. Monocytes seem to have a cellular mechanism that induces direct killing of malignant cells through cytokine-mediated cell death and phagocytosis.^[42]^ Infiltration of a large number of Treg cells into tumor tissues is usually associated with poor prognosis. More and more evidence shows that the removal of Treg cells can induce and enhance anti-tumor immune responses.^[43]^ As a component of the immune system, CD8+ T cells play an important role in suppressing tumors. CD8+ T cells can kill tumor cells with cytotoxic molecules such as granzyme and perforin.^[44]^ Macrophages exist in 2 polarization states, among which classically activated macrophages (M1) produce pro-inflammatory cytokines and reactive oxygen/nitrogen species, which are essential for host defense and tumor cell killing.^[45]^ In the C3 subtype, the increase in CD8+ T cells, M1 macrophages, and monocytes, and the decrease in Treg cells may be another potential mechanism for the better prognosis of the C3 subtype. Finally, the differential expression of immune-related genes may also be another factor for the good prognosis of C3 subtype. Compared with C1 subtype, TLR4 increased in the C3 subtype. Compared with C2 subtype, PDCD1, CTLA, LAG3 decreased, but TLR4 and SELP increased in C3 subtype. Compared with C4 subtype, ITGB2, SELP, PRF1, and TNFRSF14 increased in the C3 subtype. Compared with C5 subtype, ITGB2 and PRF1 increased in C3 subtype. Compared with C6 subtype, TNFRSF14 increased in C3 subtype. Compared with other groups, the expression of immunosuppressive factors PDCD1, CTLA, and LAG3 decreased in the C3 group,^[46,47]^ and the decreased immunostimulatory factors suggested that the C3 subtype may have a better prognosis. The differential distribution of gene mutation, immune cells, and immunoregulatory genes expression in C3 subtype relative to the other subtypes might be the mechanism leading to good prognosis in C3 subtype. Taken together, these findings highlight a means for patient stratification and targeted therapy.

However, this study has some limitations. It only used public databases for analysis, not experimental verification. The survival analyses are based on patients from the pre-immune checkpoint inhibitor (ICI) era and we do not actually know whether patients with C3 subtype respond to the ICI. The immune cell composition is inferred from bulk RNA seq and the gene expression and methylation data is a mix of all cell types in a tumor. The immune cell compositions are only reflective of a small area of tumors and given intratumoral heterogeneity and these partly reflect what is going on in these tumors as a whole.

In summary, cancer treatment has shifted from conventional strategies mainly focusing on targeted single genes to more comprehensive methods targeting the tumor microenvironment. An in-depth study of the immune status of various tumors provides valuable insights into potentially effective therapeutic strategies. Same as other studies, we characterized the recently described classification of whole-cancer immune subtypes in ccRCC and demonstrate the unique clinical and biological significance of immune subtypes in ccRCC. The biological differences observed between the immune subgroups offer avenues for the development of novel therapeutic strategies, including targeting the tumor ecosystem (including immunotherapy). The findings presented here should be taken into account when designing future treatment strategies against clear cell renal cell carcinoma.

## 5 Conclusion

Cancer is the product of a complex interaction between tumor cells and their microenvironment. Based on the infiltration pattern of 33 types of tumor immune cells, 6 immune subtypes have been previously identified. These immune subtypes offer potential treatment that may be effective regardless of tumor location or histology. However, the proportion and prognosis associated with different ISs vary by tumor type. Here, we characterized ISs in ccRCC from a clinical and molecular perspective and found that the ISs are effective indicators of ccRCC prognosis, and that IS C3 is associated with the best prognosis. High-frequency mutation genes, homologous recombination repair defects, immune cell infiltration, immune regulatory genes, and enriched signaling pathways in ccRCC display wide heterogeneity in different ISs. Their differential expression patterns suggest that different ISs may significantly impact patient survival. The biological differences observed between different ISs can be translated into heterogeneous drug responses that can be exploited to develop novel strategies (such as immunotherapy). Therefore, differences between ISs should be considered when designing future treatment strategies for improved renal clear cell carcinoma outcomes.

## Acknowledgments

The authors acknowledge the entire staff of The Cancer Genome Atlas. The results were partly based upon data generated by the TCGA Research Network (https://www.cancer.gov/tcga).

## Author contributions

**Conceptualization:** Qiang Wang, Weiting Kang, Hui Gao.

**Data curation:** Hui Gao, Zhilong Huang.

**Formal analysis:** Jinding Hu, Weiting Kang, Zhilong Huang.

**Funding acquisition:** Jinding Hu, Zhilong Huang.

**Investigation:** Jinding Hu.

**Methodology:** Jin Wang.

**Project administration:** Jin Wang.

**Resources:** Jin Wang.

**Software:** Jin Wang, Yuzhu Xiang.

**Supervision:** Hui Gao.

**Validation:** Min Fu.

**Visualization:** Min Fu.

**Writing – original draft:** Weiting Kang, Min Fu.

**Writing – review & editing:** Hui Gao, Zhilong Huang.
